# Bayesian statistical method for detecting structural and topological diversity in polymorphic proteins

**DOI:** 10.1016/j.csbj.2022.11.038

**Published:** 2022-11-21

**Authors:** Shuto Hayashi, Jun Koseki, Teppei Shimamura

**Affiliations:** Division of Systems Biology, Graduate School of Medicine, Nagoya University, Nagoya, Japan

**Keywords:** Protein structural diversity, Molecular dynamics simulation, Persistent homology, Topological data analysis, Bayesian statistical model

## Abstract

Polymorphisms in immune-related proteins and viral spike proteins are high and complicate host-virus interactions. Therefore, diversity analysis of such protein structures is essential to understand the mechanism of the immune system. However, experimental methods, including X-ray crystallography, nuclear magnetic resonance, and cryo-electron microscopy, have several problems: (i) they are conducted under different conditions from the actual cellular environment, (ii) they are laborious, time-consuming, and expensive, and (iii) they do not provide information on the thermodynamic behaviors. In this paper, we propose a computational method to solve these problems by using MD simulations, persistent homology, and a Bayesian statistical model. We apply our method to eight types of HLA-DR complexes to evaluate the structural diversity. The results show that our method can correctly discriminate the intrinsic structural variations caused by amino acid mutations from the random fluctuations caused by thermal vibrations. In the end, we discuss the applicability of our method in combination with existing deep learning-based methods for protein structure analysis.

## Graphical abstract

**Figure:**
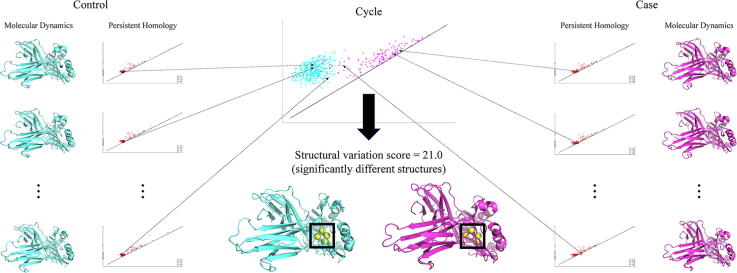

## 1 Introduction

Proteins are macromolecular compounds consisting of chains of amino acids. The amino acid sequence defines the three-dimensional structure and physical properties of the protein, and a slight difference in the amino acid sequence can alter its activity and its binding specificity and affinity to the target molecule. In particular, some immune-related proteins, such as immunoglobulins, *T*-cell receptors, and major histocompatibility complexes (MHCs), are highly polymorphic within the same species and even within the same individual. On the other hand, viral spike proteins, which interact with cell surface receptors on host cells, are also polymorphic and evolve to evade the host immune mechanism and increase the infectivity. Thus, in order to understand the immune mechanism, it is necessary to clarify how these polymorphisms lead to differences in protein structures, intramolecular and intermolecular interactions, and phenotypes.

To this end, experimental methods, including X-ray crystallography, nuclear magnetic resonance, and cryo-electron microscopy, have been widely used to solve protein structures at atomic resolution. However, there are several problems with using these experimental methods to analyze protein structural diversity. First, the experiments are conducted under conditions that are highly different from the actual cellular environment. Therefore, there is no guarantee that the protein has the same structure in a cell. Second, it is infeasible to apply the experimental methods comprehensively to various mutant proteins because they are laborious, time-consuming, and expensive. Finally, the protein structure obtained by the experimental methods is only a snapshot and does not provide information on its thermodynamic behavior in a cell.

To overcome these problems, molecular dynamics (MD) simulation can be used to obtain multiple protein conformations. In MD simulation, the trajectory of all the atoms is calculated by numerically solving Newton’s equations of motion. Since initial mutant protein structures for MD simulation can be created by substituting amino acid residues using a single template protein, there is no need to experimentally determine the structures for all the mutant proteins. Although MD simulation has greatly advanced structural biology [1], [2], [3], [4], [5], [6], [7], [8], [9], [10], [11], [12], [13], experience and expertise are required to analyze, understand, and interpret the results of MD simulation. Recently, persistent homology, a method used in topological data analysis, has been used in various types of research fields to capture the topological features of datasets [14], [15], [16], [17], [18], [19], [20], [21]. Persistent homology has the advantage of being able to quantitatively evaluate the structural information of datasets without experience or expertise. However, since previous methods use persistent homology to characterize entire protein structures, it is difficult to find differences in local substructures of mutant proteins. In fact, mutant proteins do not necessarily differ in their entire structures, but often only in their local substructures.

In this paper, we propose an in-silico approach that combines persistent homology with MD simulation to extract the topological features of proteins and detects the intrinsic variations in diverse protein substructures using a Bayesian statistical model. We applied our method to several types of HLA-DR molecules, which are one of the human MHC class II molecules, to evaluate the structural diversity. We demonstrate that our method can correctly discriminate the intrinsic structural variations caused by amino acid mutations from the random fluctuations by thermal vibrations.

## 2 Material and methods

First, three-dimensional structures of control and case proteins are created from a PDB file of a template protein. Then, MD simulations are performed to sample the protein structures that fluctuate due to thermal vibrations. Persistent homology is further applied to the sampled protein structures to capture the topological features. Lastly, differences in the topological features between the control and case proteins are scored using a Bayesian statistical model. The details are described in the following subsections.

### 2.1 Preparation of control and case protein structures

Three-dimensional structures of control and case proteins are needed to be created as inputs to MD simulations. First, a PDB file of a template protein is downloaded from the Protein Data Bank [22]. The template protein structure is then used to create the control and case protein structures by substituting amino acid residues using the Schrödinger’s Maestro’s “mutate” option [23]. For each of the control and case structures, Schrödinger’s Protein Preparation Wizard [24], [25] is used to cap N- and C-termini with acetyl and *N*-methyl groups, create disulfide bonds between close cysteine residues, and add hydrogens and optimize hydrogen bonding networks.

### 2.2 Structure sampling by MD simulation

MD simulation is used to sample the control and case protein structures. In our method, Amber 2018 [26] is used to prepare input files and perform energy minimization and MD simulation. First, a three-dimensional simulation box is created for each protein. Each protein is solvated in a TIP3P water box [27] with a buffering distance of 12 angstroms, in combination with the Amber ff99SB force field [28] and periodic boundary conditions. Na^+^ and Cl^-^ ions are also added to neutralize the charges of the system. Then, energy minimization is performed to find the stable arrangement of the atoms in the system. The stabilized structure is used as the initial coordinates of MD simulation. The simulation is performed over 100 picoseconds (500,000 time steps with a step size of 0.2 fs) while gradually heating the system from a temperature of 0 K to 310 K. The coordinates of the atoms in the system are saved at every 1,000 steps. All the 500 conformations are used in the subsequent analysis.

### 2.3 Structural feature extraction by persistent homology

**Fig. 1:**
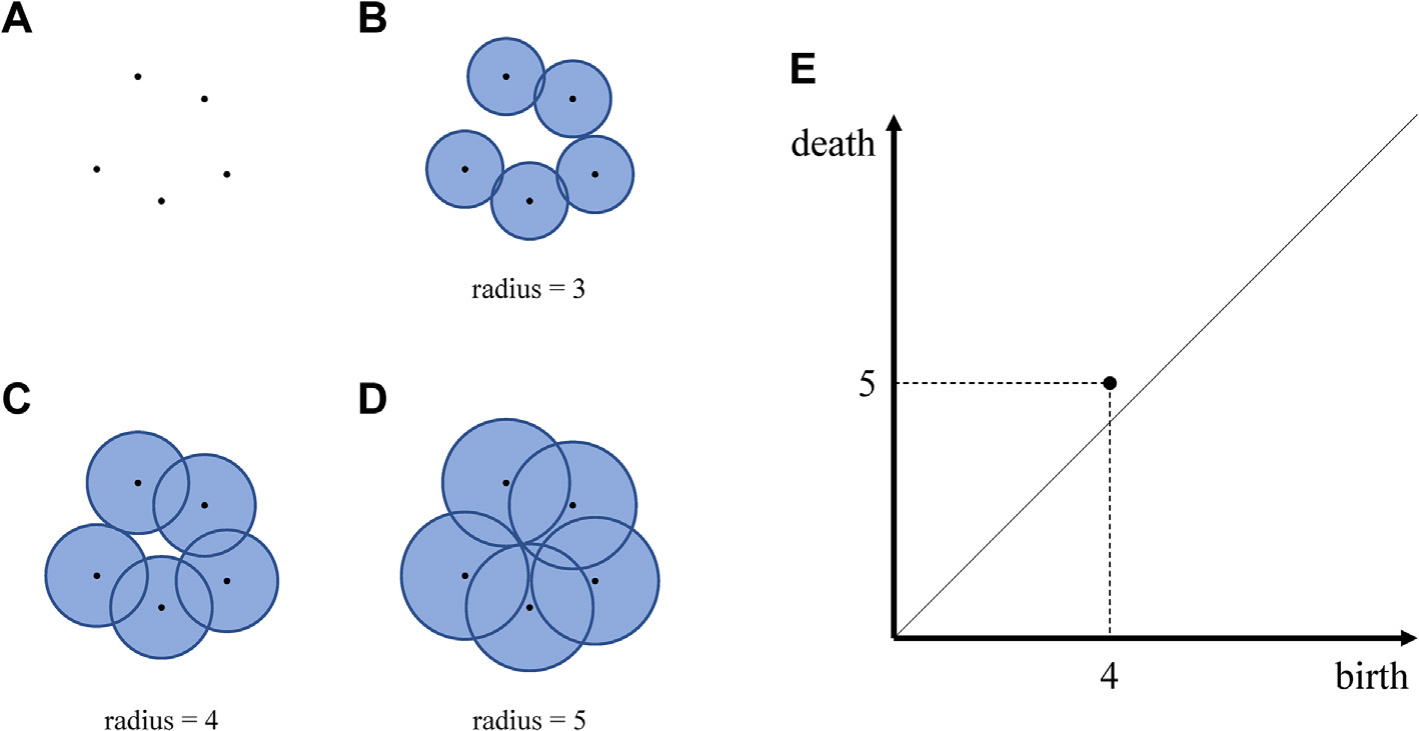
An example of the birth–death times of a loop. (A) Data points. (B) Increase in the radius of the balls. (C) Appearance of a loop. (D) Disappearance of the loop. (E) The birth–death times of the loop.

To identify the structural differences between the control and case proteins, structural features are extracted from the coordinates sampled by the MD simulations. Persistent homology can be used for this purpose, which is an approach that captures the topological properties of data points (Fig. 1A). In persistent homology, balls centered at the data points with a common radius are placed, and the radius is gradually increased (Fig. 1B). As the radius is increased, the balls intersect to form loops (1-dimensional cycles) and voids (2-dimensional cycles) (Fig. 1C). As the radius is further increased, the loops and voids disappear (Fig. 1D). These appearance and disappearance times of the cycles, called birth and death times, reflect the topological characteristics of the data points and are used to analyze the shape formed by the data points (Fig. 1E).

First, the coordinates of alpha carbons are extracted from the sampled protein structures to obtain their backbone structures. To focus on important structural variations, alpha carbons in intrinsically disordered regions are identified by the DSSP algorithm [29] and excluded. Persistent homology is then applied to the remaining alpha carbons using the TDA package in R [30], which produces a time series of the birth and death times for each cycle of each protein. Since each cycle is not created for all the conformations, the number of obtained birth and death times could be less than 500. In addition, a cycle observed in the control protein is not necessarily observed in the case protein, and vice versa. Therefore, only cycles that satisfy the following two conditions are used in the subsequent analysis: (i) the cycle is obtained at least five times for both proteins, and (ii) the cycle is obtained within the first five steps for both proteins.

### 2.4 Detection of structural variations between control and case proteins

The time series of the birth and death times of a given cycle indicates how the atoms that make up the cycle thermally vibrate. To detect the structural variations between the control and case proteins, intrinsic structural variations must be distinguished from thermal vibrations. Furthermore, the thermal vibrations might not be fully captured when the number of obtained birth and death times is insufficient. To address these problems, we propose a method to identify intrinsic structural variations based on a Bayesian statistical model.

For each cycle, let $\left(b_{t}^{\left(1\right)},d_{t}^{\left(1\right)}\right)$ and $\left(b_{t}^{\left(2\right)},d_{t}^{\left(2\right)}\right)$ be the birth and death times at time t for the control and case proteins. For simplicity, the subscript for the cycle is omitted. To reduce the correlation that the death time is nearly equal to the birth time, the lifespans $l_{t}^{\left(1\right)}=d_{t}^{\left(1\right)}-b_{t}^{\left(1\right)}$ and $l_{t}^{\left(2\right)}=d_{t}^{\left(2\right)}-b_{t}^{\left(2\right)}$ are used instead of the death times. Let $x_{t}^{\left(1\right)}={\left(b_{t}^{\left(1\right)},l_{t}^{\left(1\right)}\right)}^{T}$ and $x_{t}^{\left(2\right)}={\left(b_{t}^{\left(2\right)},l_{t}^{\left(2\right)}\right)}^{T}$, then the observations are generated from the following hierarchical Bayesian statistical model: For each p∈{1,2},

$$\Sigma^{\left(p\right)}\;∼\;W^{-1}\left(\Psi_{0},\;\nu_{0}\right),$$

$$\mu^{\left(p\right)}\;|\;\Sigma^{\left(p\right)}\;∼\;N\left(m_{0},\;\frac{\Sigma^{\left(p\right)}}{\kappa_{0}}\right),$$

$$x_{t}^{\left(p\right)}\;∼N\left(\mu^{\left(p\right)},\Sigma^{\left(p\right)}\right),$$

where $W^{-1}$ is an inverse Wishart distribution, N is a normal distribution, and $\Psi_{0}\in R^{2\times 2}$, $\nu_{0}\in R$, $m_{0}\in R^{2}$, and $\kappa_{0}\in R$ are constant parameters that control the prior distributions. Here, we set $\Psi_{0}$ to the identity matrix and $\nu_{0}$ to 2 so that the inverse Wishart distribution is a non-informative prior. Similarly, we set $\kappa_{0}$ to +0 so that the normal distribution is also a non-informative prior. In this case, the normal distribution becomes an improper prior, but it is guaranteed to have a proper posterior because the birth and death times are obtained at least five times. The values of $m_{0}$ do not affect the posterior distribution because $\kappa_{0}$ is +0.

Since the prior distributions are conjugate, the posterior distributions of $\mu^{\left(p\right)}$ and $\Sigma^{\left(p\right)}$ can be analytically calculated as follows:

$$\Sigma^{\left(p\right)}\;|\;{\left\{x_{t}^{\left(p\right)}\right\}}_{t}∼W^{-1}\left(\Psi^{\left(p\right)},\;\nu^{\left(p\right)}\right),$$

$$\mu^{\left(p\right)}\;|\;\Sigma^{\left(p\right)},{\left\{x_{t}^{\left(p\right)}\right\}}_{t}\;∼N\left(m^{\left(p\right)},\frac{\Sigma^{\left(p\right)}}{\kappa^{\left(p\right)}}\right),$$

where

$$\Psi^{\left(p\right)}=\Psi_{0}+S^{\left(p\right)},$$

$$\nu^{\left(p\right)}=\nu_{0}+N^{\left(p\right)},$$

$$m^{\left(p\right)}={\overset{¯}{x}}^{\left(p\right)},$$

$$\kappa^{\left(p\right)}=N^{\left(p\right)},$$

and

$$N^{\left(p\right)}=\left|{\left\{x_{t}^{\left(p\right)}\right\}}_{t}\right|,$$

$${\overset{¯}{x}}^{\left(p\right)}=\frac{1}{N^{\left(p\right)}}\sum_{t}x_{t}^{\left(p\right)},$$

$$S^{\left(p\right)}=\sum_{t}\left(x_{t}^{\left(p\right)}-{\overset{¯}{x}}^{\left(p\right)}\right){\left(x_{t}^{\left(p\right)}-{\overset{¯}{x}}^{\left(p\right)}\right)}^{T}.$$

The posterior distributions of $\mu^{\left(p\right)}$ and $\Sigma^{\left(p\right)}$ represent the extent to which the thermal vibrations cause the cycle to fluctuate in the protein.

To detect the structural variations between the control and case proteins, $\mu^{\left(1\right)}$’s, $\Sigma^{\left(1\right)}$’s, $\mu^{\left(2\right)}$’s, and $\Sigma^{\left(2\right)}$’s are sampled from the posterior distributions and compared. If the cycle observed in the control protein is similar in structure to the case protein, the distributions for the control protein $N(\mu^{\left(1\right)},\Sigma^{\left(1\right)})$ and the case protein $N(\mu^{\left(2\right)},\Sigma^{\left(2\right)})$ should be almost the same. Conversely, If the cycle observed in the control protein has an intrinsically different structure from the case protein, $N(\mu^{\left(1\right)},\Sigma^{\left(1\right)})$ and $N(\mu^{\left(2\right)},\Sigma^{\left(2\right)})$ would be dissimilar. To measure the similarity of the distributions, the Jeffreys divergence [31] is used. The Jeffreys divergence of two probability distributions p and q is defined by

$$D_{J}\left(p,q\right)=D_{\mathrm{KL}}\left(p||q\right)+D_{\mathrm{KL}}\left(q||p\right),$$

where $D_{\mathrm{KL}}$ is the Kullback-Leibler divergence [32] and given by

$$D_{\mathrm{KL}}\left(p||q\right)=\int p\left(x\right)\log\frac{p\left(x\right)}{q\left(x\right)}dx.$$

The Jeffreys divergence is larger than or equal to 0, and similar two distributions have a small Jeffreys divergence. The Jeffreys divergence of two normal distributions can be analytically calculated because the Kullback-Leibler divergence of two normal distributions is also analytically given by

$$D_{\mathrm{KL}}\left(N\left(\mu_{1},\Sigma_{1}\right)\left|\right|N\left(\mu_{2},\Sigma_{2}\right)\right)=\frac{1}{2}\left(\log\frac{\left|\Sigma_{2}\right|}{\left|\Sigma_{1}\right|}-d+tr\left(\Sigma_{2}^{-1}\Sigma_{1}\right)+{\left(\mu_{2}-\mu_{1}\right)}^{T}\Sigma_{2}^{-1}\left(\mu_{2}-\mu_{1}\right)\right),$$

where d is the dimension of the normal distributions. Thus, the Jeffreys divergences can be calculated from each of the sampled $\mu^{\left(1\right)}$’s, $\Sigma^{\left(1\right)}$’s, $\mu^{\left(2\right)}$’s, and $\Sigma^{\left(2\right)}$’s.

Since the parameters are sampled multiple times, the Jeffreys divergences can also be calculated multiple times. The structural variation in the cycle between the control and case proteins is detected using a score defined by the 5th percentile of the calculated Jeffreys divergences, which we call a structural variation score (SV score). If the SV score is larger than a certain threshold $D^{*}$, the Jeffreys divergences are determined to be significantly larger than 0. This means that the distributions of the birth and death times are different between the control and case proteins, and that these two proteins have different structures in the cycle. In our method, we set $D^{*}$ to 4.

## 3 Results

### 3.1 Application of our method to HLA-DR complexes

**Fig. 2:**
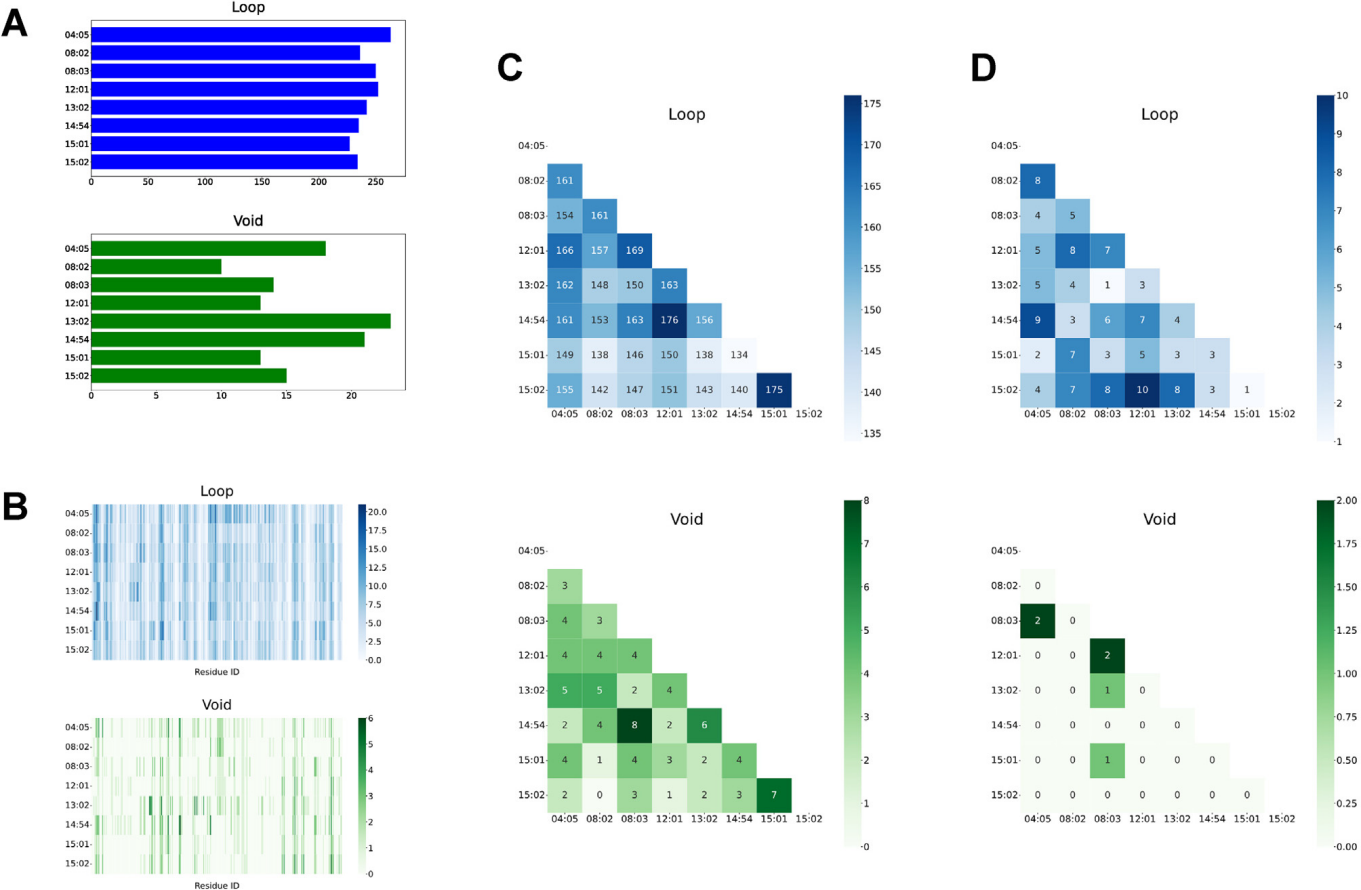
Statistics of cycles observed in the HLA-DR complexes. (A) The number of cycles observed in each complex. (B) The number of cycles consisting of each amino acid residue in each complex. (C) The number of cycles observed in both complexes of each combination. (D) The number of cycles in which structural variations were detected by our method.

We applied our method to HLA-DR molecules to demonstrate its capability. First, we downloaded an X-ray crystal structure of the HLA-DRA1*01:01 and HLA-DRB5*01:01 complex (PDB ID: 1H15) to be used as a template. By replacing amino acid residues in the template beta chain, we created eight mutant HLA-DR complexes (DRB1*04:05, DRB1*08:02, DRB1*08:03, DRB1*12:01, DRB1*13:02, DRB1*14:54, DRB1*15:01, and DRB1*15:02), which are common in Japanese. We performed MD simulation and persistent homology for these complexes as described in Material and Methods to obtain cycles. Fig. 2A shows the number of cycles observed in each complex, and Fig. 2B shows how many cycles each residue formed, which indicates that several regions tend to form cycles across the complexes. We then applied our Bayesian statistical model to each combination of the complexes. Fig. 2C shows the number of cycles observed in both complexes of each combination, and Fig. 2D shows the number of cycles in which structural variations were detected by our model. Below, we will show several examples of structural variations captured by our method.

### 3.2 Loop variation detected across HLA-DR complexes

**Fig. 3:**
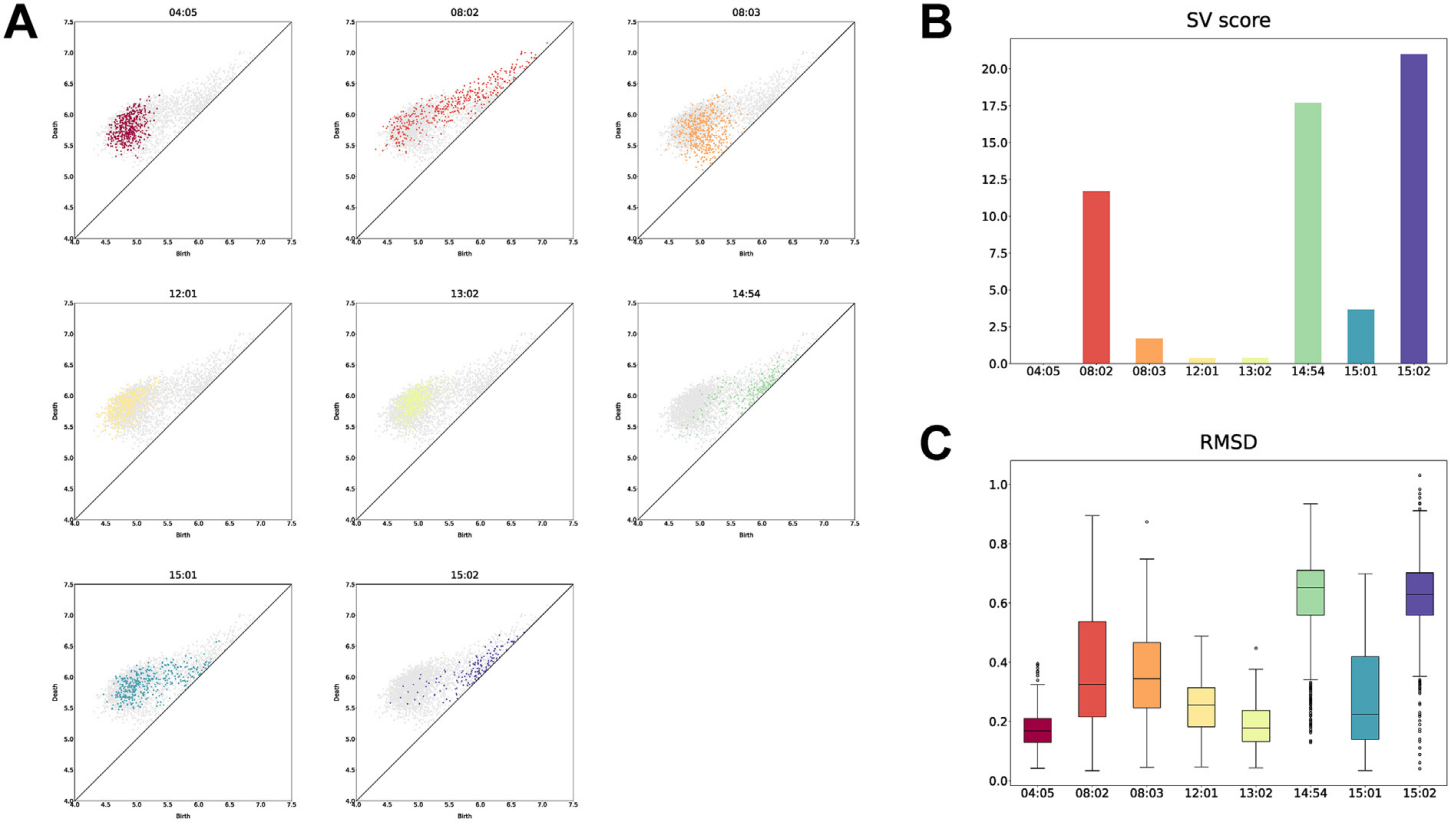
A loop in which the structural variation was detected across the HLA-DR complexes. (A) The scatter plots of the birth and death times of the loop in each HLA-DR complex. (B) The SV scores of the loop calculated using the DRB1*04:05 complex as control and the other complexes as cases. (C) The RMSDs between the average loop structure of the DRB1*04:05 complex and the loop conformations of the eight complexes.

First, we show a loop in which the structural variation was detected across the complexes. Fig. 3A shows the scatter plots of the birth and death times of the loop. This figure implies that the complexes can be classified based on the topological similarity of the loop: cluster1 (the DRB1*04:05, DRB1*12:01, and DRB1*13:02 complexes) and cluster 2 (the DRB1*14:54 and DRB1*15:02 complexes). Although the DRB1*08:02 complex has a similar structure to cluster 1, it has larger birth times. The loop structures in the DRB1*08:02 and DRB1*15:01 complexes fluctuate and move between the two clusters. We calculated the SV scores using the DRB*04:05 complex as control and the other complexes as cases (Fig. 3B). The SV scores for the DRB1*08:02, DRB1*14:54, and DRB1*15:02 complexes were higher than the criterion $D^{*}=4$, while the SV scores for the DRB1*12:01 and DRB1*13:02 complexes were smaller compared to the other SV scores, which means that our scoring method can correctly distinguish topological clusters. We also checked the root-mean-square deviations (RMSDs) between the average loop structure of the DRB1*04:05 complex, which was obtained by extracting and superimposing the loop conformations of the DRB1*04:05 complex and taking the average of the coordinates, and the loop conformations of the eight complexes (Fig. 3C). Comparison of Fig. 3B and 3C suggests that the SV scores are correlated with the RMSDs.

### 3.3 Loop variation detected between similar complexes

**Fig. 4:**
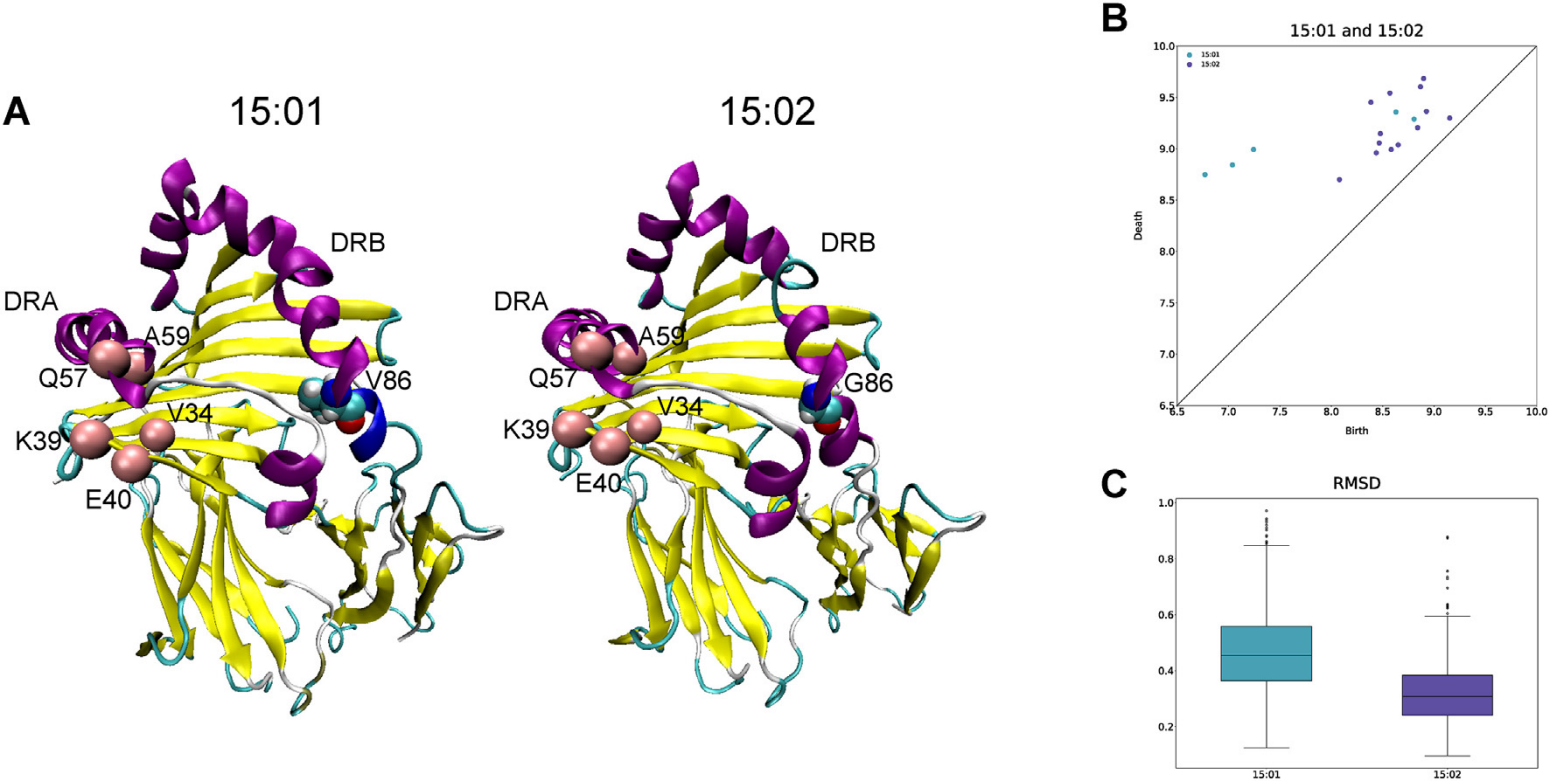
The only loop in which the structural variation was detected between the DRB1*15:01 and DRB1*15:02 complexes. (A) The structures of the two complexes. (B) The scatter plot of the birth and death times of the loop in the complexes. (C) The RMSDs between the average loop structure of the DRB1*15:02 complex and the loop conformations of the two complexes.

Next, we show the only loop in which the structural variation was detected between the DRB1*15:01 and DRB1*15:02 complexes, which have the same amino acid sequence except that the 86th amino acid residue is a valine and a glycine, respectively (Fig. 4A). This loop consists of five amino acid residues in the alpha chain DRA*01:01 (V34, K39, E40, Q57, and A59) located near the 86th amino acid residue of the beta chain. It has been reported that the 86th amino acid residue of the beta chain affects the stability of the HLA-DR complex [33], suggesting that it is reasonable that the mutation in the beta chain induces the structural variation in the alpha chain. Furthermore, because the loop is located at the peptide-binding groove, the structural change could alter the peptide-binding motif. Fig. 4B shows the scatter plot of the birth and death times of the loop. We also checked the RMSDs between the average loop structure of the DRB1*15:02 complex and the loop conformations of the two complexes (Fig. 4C). Although the loop was observed only 5 and 13 times in the DRB1*15:01 and DRB1*15:02 complexes, respectively, our method was able to detect the structural variation by using the Bayesian statistical model.

To demonstrate the effectiveness of our method, we checked whether structural differences in DRB1*15:01 and DRB1*15:02 can be detected by RMSDs alone. We compared the average structures between the two complexes and found no significant difference (RMSD = 0.744Å). We also found that the structural fluctuations were small in the two complexes (standard deviations of RMSDs = 0.097Å and 0.063Å for DRB1*15:01 and DRB1*15:02, respectively). Thus, there is no significant difference in the entire structures between DRB1*15:01 and DRB1*15:02, which makes it difficult to find substructural differences in these two complexes using RMSDs alone.

### 3.4 Void variation detected among several complexes

**Fig. 5:**
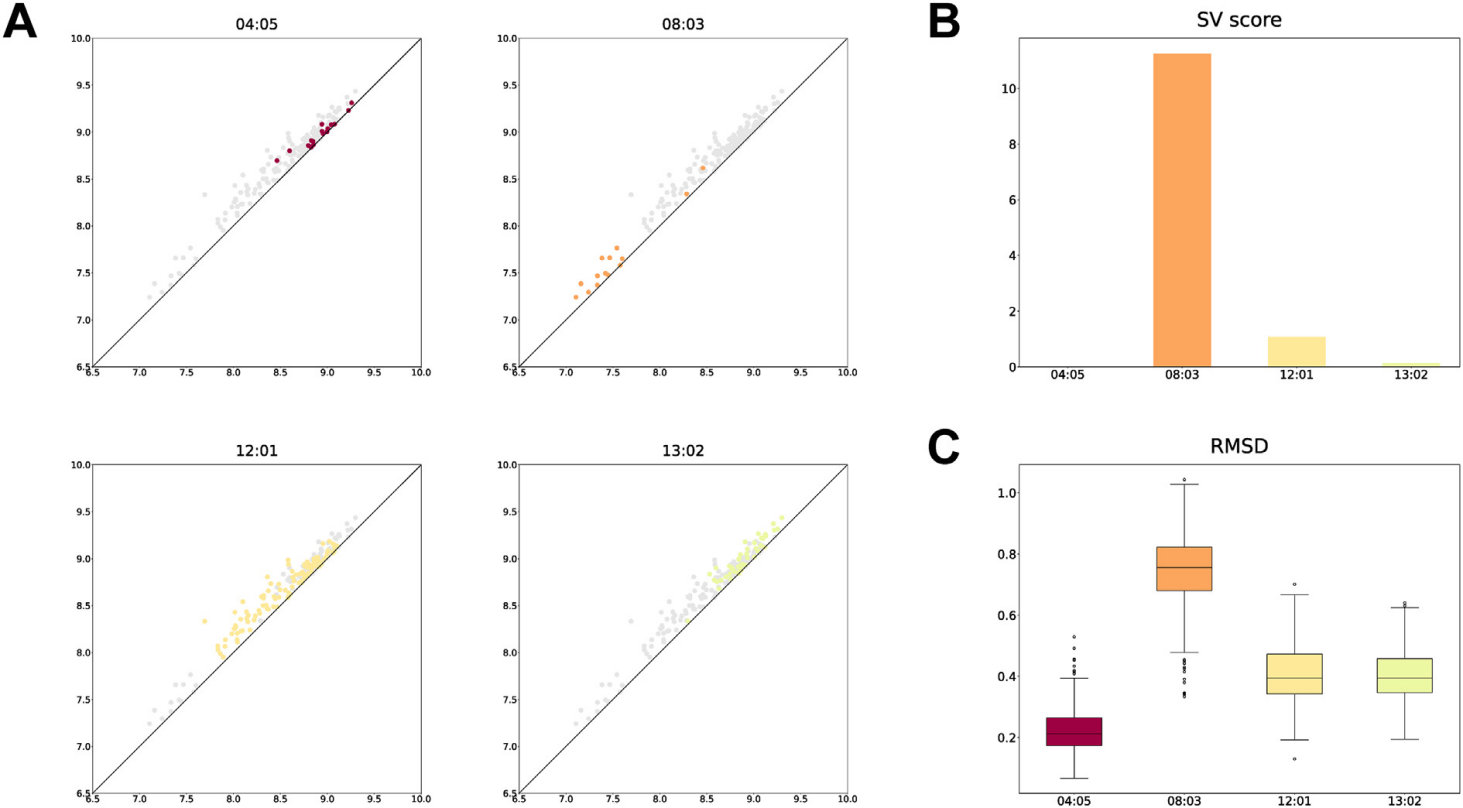
A void in which the structural variation was detected among the DRB1*04:05, DRB1*08:03, DRB1*12:01, and DRB1*13:02 complexes. (A) The scatter plots of the birth and death times of the void in each of the four complexes. (B) The SV scores of the void calculated using the DRB*04:05 complex as control and the other complexes as cases. (C) The RMSDs between the average void structure of the DRB1*04:05 complex and the void conformations of the four complexes.

Finally, we show a void in which the structural variation was detected among the DRB1*04:05, DRB1*08:03, DRB1*12:01, and DRB1*13:02 complexes. Fig. 5A shows the scatter plots of the birth and death times of the void, which suggests that the DRB1*08:03 complex has a different structure from the other complexes. We calculated the SV scores using the DRB*04:05 complex as control and the other complexes as cases (Fig. 5B). As expected, only the SV score for the DRB1*08:03 complex was higher than the criterion $D^{*}=4$. We also checked the RMSDs between the average void structure of the DRB1*04:05 complex and the void conformations of the four complexes (Fig. 5C). Since the SV scores are correlated with the RMSDs, it can be said that our method can capture structural variations characterized not only by loops but also voids.

## 4 Discussion

In this paper, we proposed a method for detecting protein structural variations using MD simulations, persistent homology, and a Bayesian statistical model. Our method has several advantages. First, our method uses multiple protein structures sampled by MD simulation rather than a single snapshot of the protein structure. Therefore, it is possible to determine whether the difference between two protein structures is intrinsic or due to thermal vibrations. Second, our method is quantitative using persistent homology and does not require experience or expertise in protein structure analysis. Third, our method can correctly identify structural variations in cycles, even when they were observed only several times. This is because our method can measure the credibility of the inference result by sampling the parameters and the Jeffreys divergences in our Bayesian statistical model. Finally, our method focuses on differences in the minimal structural components, such as loops and voids, rather than differences in the coordinates of atoms. Hence, our method has the potential to efficiently extract substructural variations that may alter the functions.

Although the threshold for SV scores was set to 4 in this study, it needs to be chosen depending on how different substructures should be detected because SV scores reflect the magnitudes of structural differences rather than statistical significances. Theoretically, if the two covariance matrices of birth–death times are equal between control and case proteins ($\Sigma_{1}=\Sigma_{2}=\Sigma$), then the SV score is equal to the square of the Mahalanobis' distance between the two means (${\left(\mu_{2}-\mu_{1}\right)}^{T}\Sigma^{-1}\left(\mu_{2}-\mu_{1}\right)$). In this study (SV threshold = 4), this implies that the two means are more than 2 S.D. apart. By using a larger SV threshold, only substructures with large differences can be detected.

Recent advances in deep learning technology have enabled highly accurate biomolecular structural analysis in silico, such as protein structure inference [34], [35], [36], [37], molecular conformation generation [38], [39], [40], and protein–ligand docking [41], [42], [43]. A large amount of structural information on biomolecules can be easily obtained, and structural biology has been greatly influenced. By combining with the technology, our method would help accelerate this research field elucidate the biological meanings of protein diversities.

## CRediT authorship contribution statement

**Shuto Hayashi:** Conceptualization, Methodology, Software, Writing – original draft, Funding acquisition. **Jun Koseki:** Conceptualization, Formal analysis, Writing – review & editing, Funding acquisition. **Teppei Shimamura:** Writing – review & editing, Supervision, Funding acquisition.

## Declaration of Competing Interest

The authors declare that they have no known competing financial interests or personal relationships that could have appeared to influence the work reported in this paper.
